# A complex wireless sensors model (CWSM) for real time monitoring of dam temperature

**DOI:** 10.1016/j.heliyon.2023.e13371

**Published:** 2023-02-01

**Authors:** Jamil Afzal, Zhou Yihong, Usama Afzal, Muhammad Aslam

**Affiliations:** a College of Hydraulic & Environmental Engineering, China Three Gorges University, Yichang, 443002, China; b School of Microelectronics, Tianjin University, Tianjin, China; c Department of Statistics, Faculty of Science, King Abdulaziz University, Jeddah, Saudi Arabia

**Keywords:** Real time, Dam temperature, Complex wireless sensor model, CWSM

## Abstract

The following work is based on real-time temperature monitoring during the construction and during the operation of a dam. For this purpose, we have proposed a sensing model named: the “complex wireless sensors model (CWSM)” for measuring the value of different factors like temperature, humidity and pressure on the dam. The installation of the proposed model has been discussed with its wireless networking. The model contains five types of sensors i.e. humidity, temperature, pressure, sun and irradiance sensors for measuring the variation of different factors. The computation modeling of CWSM has been discussed in the paper. 3-D Finite Element method is used for thermal analysis. As the result, it is concluded that the wireless network will be more suitable for measuring and analyzing the effects of temperature, humidity, water pressure and solar radiation on the dam.

## 1 Introduction

In the engineering of concrete dams, crack management is the main consideration. Because a large proportion of concrete splints are caused by changes in temperature, temperature management is the main strategy for crack prevention [1]. Considerable sums of concrete are often used to produce a solid large concrete structure during the building of a concrete dam. The pressure generated by the hydration of cement becomes fond of in the structural element, just because of its sheer pressure magnitude, a concrete dam took many years to reach a uniform temperature after reaching its maximum temperature [2]. Such weather patterns in concrete dams tend to result in cracks and swear breakages, which also have a serious influence on the dam’s safety and smooth functioning. As a consequence, fracture mitigation is critical during the design of concrete dams. Many researchers have undertaken studies into the mechanism of typical temperature cracking and the impacts of different heating control strategies in order to prevent such cracking. The groundwork for confronting the problem of temperature management and fracture prevention understands the true temperature distribution of the concrete dam. Many complex technological challenges must be handled during the building of such gigantic dams. Due to absorption heat, cooling circumstances, challenging exterior surroundings, and strong foundation restrictions, temperature management and fracture control are the key technical challenges in the initial design and construction phase of mass concrete buildings [3]. Years of industrial expertise and extensive study have resulted in a set of anti-cracking methods. Even yet, if the true temperature variation of the material can also be determined in a timely way, it is impossible to develop a good temperature management strategy.

The monitoring system of concrete dam is becoming a noticeable problem in today’s world, and it involves pursuing the constant and inconstant characteristics of large hydroelectric dams. Temperature is among the most critical factors that influences the kinematics ductility of concrete dams, particularly. As a result, deliberate the natural climatic patterns in concrete dam is actual explanatory, as they influence thermal heating, which may produce high loads in concrete dam walls and have a substantial impact on the formation and conduct of fractures, as well as deflections and global climate change which might leads to an over-all increase in the temperature of these structures of composite dams [4]. The temperature has a significant impact on the permanent reinforcement of concrete dams, in the areas of thermal analysis linked to non-linear and non-heat flux through a solid, taking into account appropriate boundary conditions. Finite Element Method (FEM) – based approach for determining periodic temperature and stress patterns in concrete are used in dams [5]. The heating characteristics of concrete dam constructions has really been considerably better explored using this method, both in the dam initial system design and in the particular phase of dam management and control. It has been addressed in recent rising researches relating with: “*Stress Analysis of Concrete Structures*” that are adversely affected by parameter of thermal stress [6]; the tenacity of the regular intervals heat flux in a concrete dam [7]; and the determination of the relatively frequent temperature distribution in a concrete dam. The influence of environmental vulnerability ramification on thermal stress analysis of the mentioned arch concrete dam [8], the thermal and stress analysis of discussed roller-compacted concrete dam [9]; the numerical calculations of temperature in a mass concrete dam [4]. More information can be seen in Refs. [10,11].

Other studies have identified the founding of innovative computational methods, including the framework for the analysis of concrete dams related to ecological thermal effects, the hydrostatic pressure and temperature time-displacement model for concrete dams, and the hydrostatic pressure cyclical state model for concrete dam observing analysis of the data [12]. Different studies are also conducted to find the effect of dam on temperature of surrounding area [1]. Some others have discussed the sensitivity analysis of thermal field in enormous structures and the temperature directive consideration of concrete dams in arctic regions [12]. In contemporary times, a significant volume of study has been conducted on the temperature comportment of concrete dams, considering the effects of implementing environmental parameters. Some researchers have been focusing on detailed studies of displacements involving: analysis of concrete dams based on seasonal hydrostatic damage due to static load, seasonal thermal displacements of gravity dams in cold regions, the method of dam deformation due to thermal actions, and the correlation between atmospheric temperature and daily fluctuation of structural performance [12]. Others have looked at dam structural fractures, while others have looked into temperature-induced pressures in concrete dams. Many have researched shadowing implications and radiation from the sun impacts, even though other investigation have explored thermal properties in extremely dams [13].

The following search work has described the real-time monitoring of a dam temperature. A prototype wireless sensing model has been proposed in this work. Four types of sensors have been utilized in this model, which uses wireless networking for communication. Through this model, one can be able to monitor the effects of the temperature, humidity, water pressure and sun elevation on the dam. Also, for the analysis of the thermal analysis 3-D, the Finite Element method is used.

## 2 Complex wireless sensors model (CWSM)

**Fig. 1:**
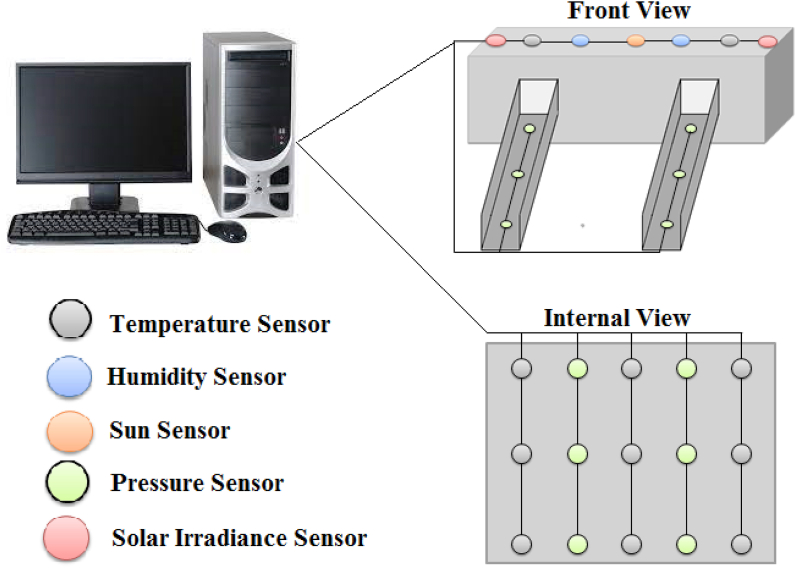
Sensor installation in the dam.

As for the real-time monitoring of the dam temperature, we have proposed a complex wireless sensors model (CWSM). The CWSM is based on four types of wireless sensors which will be used for the measuring the effect of temperature, humidity and water pressure on the dam and the elevation of sun. According to best, generally a dam is affected by temperature, humidity and water pressure. Some-times sun elevation also affects dam. That’s why we have chosen above four types of sensors. One can easily observe and measure the effect of above factors. The complex wireless sensors model installation is showing in Fig. 1.

All the sensors are installed at specific distance. For example the internal side of dam contains two types of sensor i.e. temperature and pressure. Through these sensors, we want to find the temperature and pressure effects of reservoir on the dam. Similarly, temperature, humidity and solar irradiance sensors are implanted at the top of the dam. The temperature sensors will be used to observe the temperature effects of sun on the dam. Moreover, humidity sensors will be used for humidity effects and sun sensors to measure the sun elevation. Also the pressure sensors will be implanted in the tunnels to measure pressure of water flow. One can also implanted pressure sensor at the spillway gate to measure the pressure of water flows from spillway.

### 2.1 Networking of model

**Fig. 2:**
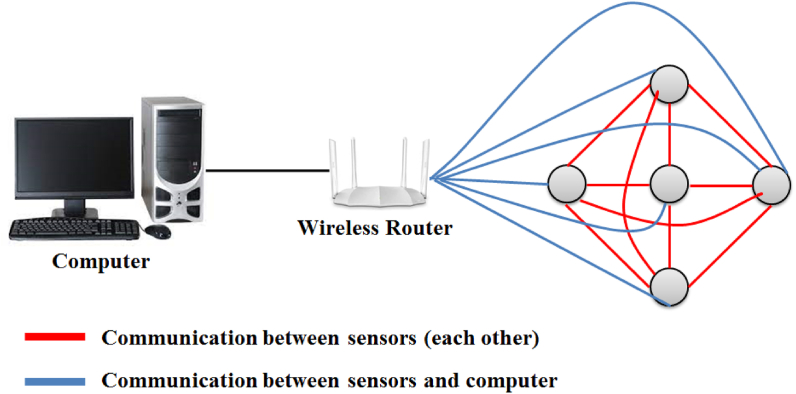
An over view of wireless communication between sensors.

Our model has recommended the wireless networking [14] instead of wire or optical connection. But electric wire will be implanted with the sensors to provide the electricity for charging purposes. However, data communication will be performed through the wireless networking. All sensors will not only connect with computer but also with each other as shown in Fig. 2. If something happened with one sensor, the surrounding sensors will automatically detect the problem and will raise the alarm. So that each sensor will has decision making capability [15]. For example, if one sensor cut off from the power supply, the nearest sensors will raise the alarm. In this way, one will easy able to identify that sensor and will repair without losing time.

If we talk about the sensors the fabrication or best material for the sensors, according to our best graphene (due to novel structure and other properties [16]) based temperature [17,18], pressure [19] and humidity [20] sensors are more effective than other materials. Future more, if we talk about sensors data analysis one can use different statistic approaches as we have worked in our previous works [[21], [22], [23]].

### 2.2 Sensors

The model is based on four types of sensors i.e. temperature, humidity, pressure and solar irradiance sensors. Let us see some over view of sensors.

#### 2.2.1 Temperature sensor

**Fig. 3:**
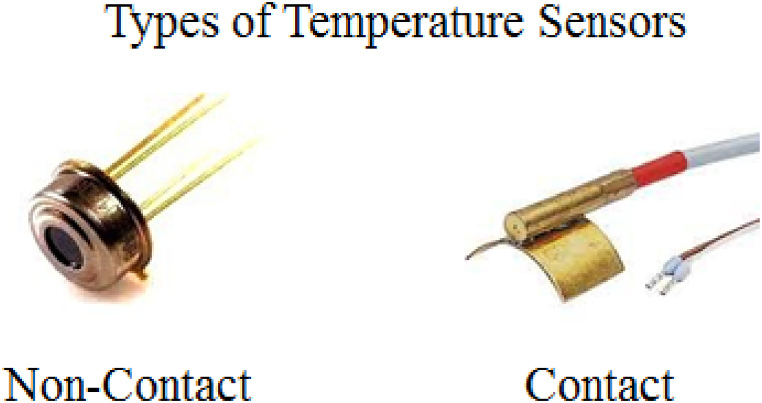
Types of temperature sensors.

Temperature sensors are widely used sensor in our daily life for different purposes like to monitor the temperature of dam, concrete [24] and others. A temperature sensor is an electric resistance temperature detector that gives the measurement of temperature from its surrounding in the form of electrical signals [25,26]. Numbers of researchers have work on fabrication of temperature sensor as can be seen in following references [17,27,28]. There are two types of temperature sensor i.e. contact and non-contact temperature sensors as showing in Fig. 3. Contact temperatures are directly connected with the body and measure the temperature in degree of hotness and coldness directly [29]. Similarly, Non-contact temperature sensors are not connected with body and measure the hotness and coldness of body from radiation emission [30].

#### 2.2.2 Pressure sensor

**Fig. 4:**
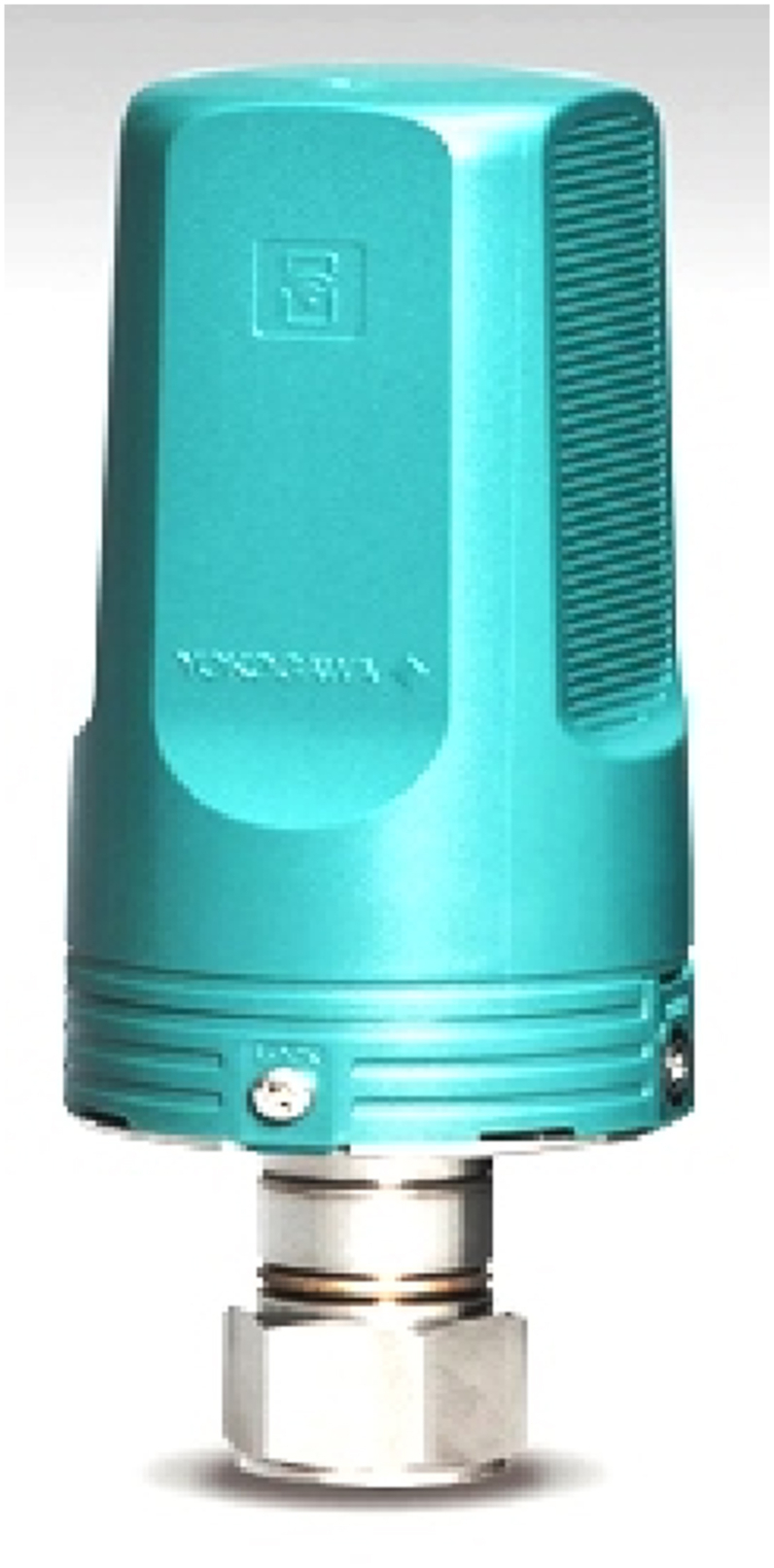
Wireless pressure sensor.

Pressure sensors are widely used sensor in our daily life for different purposes like to monitor the flow of gas, water level and speed [[31], [32], [33]] etc. A pressure sensor is an electronic device used to measure the pressure of exerted by liquid and gases [34] as shown in Fig. 4. All sensing element of pressure sensor react against the pressure and produce the electric signals, which can be read on computer or laptop [35]. Different materials are used in the fabrication of pressure sensors as can be seen in following references [[36], [37], [38], [39]].

#### 2.2.3 Humidity sensor

**Fig. 5:**
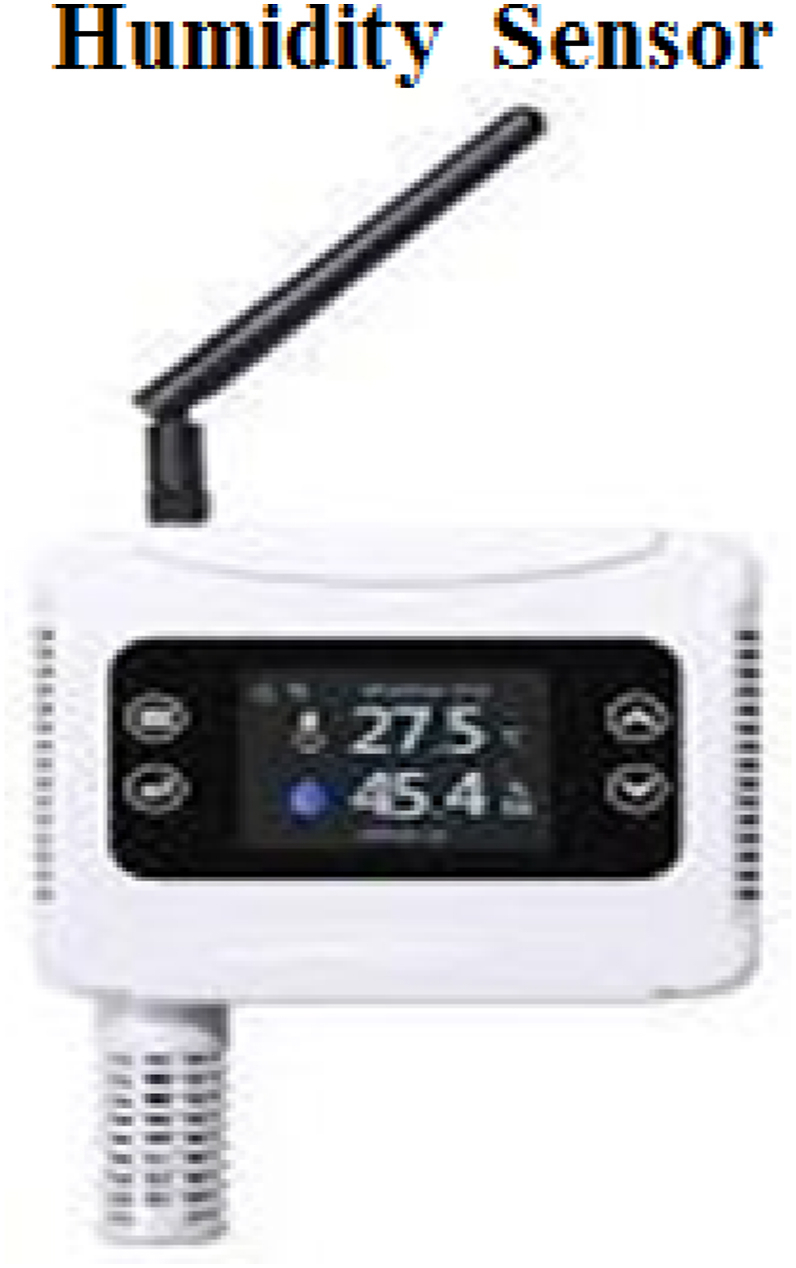
Wireless humidity sensor.

Humidity sensors are widely used sensor in industry to measure and control the humidity in the atmosphere [40]. The humidity sensor is an electric device that senses, measures, and reports the relative humidity (RH) of air or determines the amount of water vapor present in gas mixture (air) or pure gas and produced electric signals [41] as shown in Fig. 5. For the fabrication one can concern the following references [21,[42], [43], [44]].

#### 2.2.4 Sun sensor

**Fig. 6:**
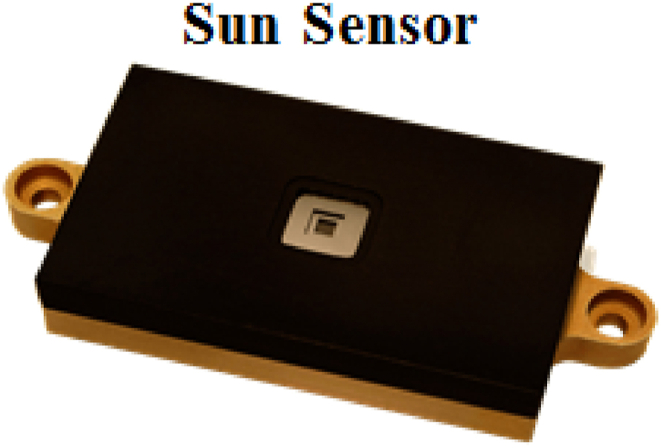
Sun sensor.

Sun sensor is used to detect the position of the sun [45] as shown in Fig. 6. The fabrication of sun sensor can be seen in following references [[46], [47], [48]].

#### 2.2.5 Solar irradiance sensor

**Fig. 7:**
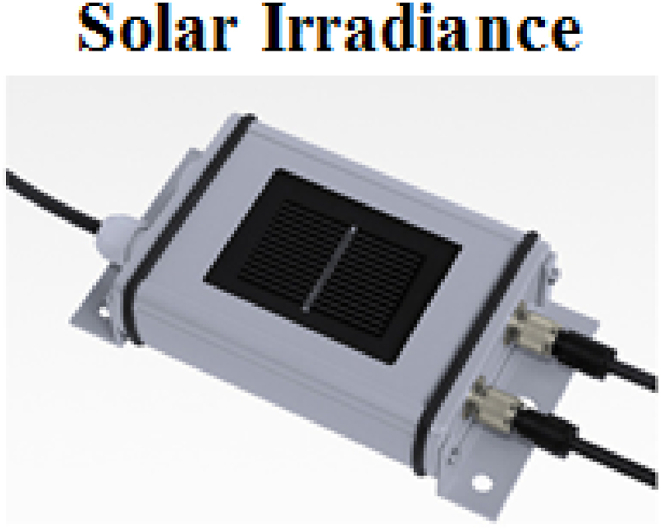
Wireless solar irradiance sensor.

Solar irradiance sensor is commercially used to determine the solar radiations [49] as shown in Fig. 7. The sun radiations are absorbed by the sensor and sensor produces the readable electric signals. The fabrication of sensor can be seen in following references [[50], [51], [52]].

## 3 Modeling of CWSM

**Fig. 8:**
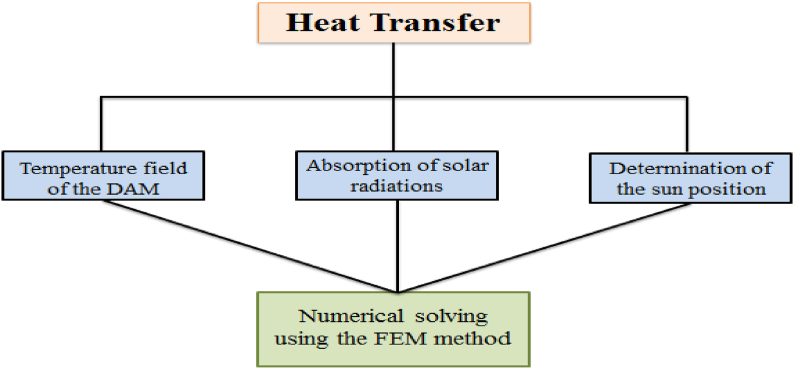
Flow chart of heat transfer of CWSM.

Let us move to the modeling of CWSM as shown in Fig. 8. The figure is showing the modeling of the heat transfer of the proposed model.

### 3.1 Temperature field of the dam

Following heat transfer equation is used for the filed temperature, by assume that homogeneous, isotropic solid, thermal conductivity is independent of temperature and variable boundary conditions as can be seen in Eq. (1).

(1) $$\frac{\partial^{2}T}{{\partial x}^{2}}+\frac{\partial^{2}T}{{\partial y}^{2}}+\frac{\partial^{2}T}{{\partial z}^{2}}=\frac{\rho c}{\lambda }\frac{\partial T}{\partial t}$$

whereas T is temperature (K), x y z are Cartesian coordinates (m), $Whereas\;T\;is\;temperature\;\left(K\right),x\;y\;z\;are\;Cartesian\;coordinates\;\left(m\right),\rho density\;\left(kg/m^{3}\right),c\;is\;specific\;heat\;\left(J/\left(kg\;K\right)\right),\lambda \;is\;thermal\;conductivity\;\left(W/\left(m\;K\right)\right)\;and\;t\;is\;time\left(s\right)\text{.}$

### 3.2 Absorption of solar radiation

We use Energy flux density (Dilger et al., 1983) for absorption of solar radiation, as can be seen in Eq. (2)

(2) $$q_{s}=Iacos\theta_{s}$$

whereas $q_{s}$ is energy flux density of solar radiation absorbed by the solid (W/m^2^), I is energy flux density of solar radiation absorbed at Earth’s surface (W/m^2^), a is solar absorptivity of surface and $\theta_{s}$ is angle of incidence of Sun’s rays (^o^), as can be seen in Eq. (3).

(3) $$I=I_{sc}k_{T}$$

$I_{sc}$ is solar constant (on average 1350 W/m^2^) and $k_{T}$ is transparency factor, which depends on atmospheric weather conditions and the path length of the radiation through the atmosphere, as can be seen in Eq. (4).

(4) $${\cos\;\theta }_{s}=-{\cos\;\delta }_{s}\;\cos\;\Omega \;\sin\;\alpha \;{\cos\;\tau }_{s}\;\sin\;\Phi +{\cos\;\delta }_{s}\;\sin\;\Omega \;\sin\;\alpha \;{\sin\;\tau }_{s}+{\sin\;\delta }_{s}\;\cos\;\Omega \;\sin\;\alpha \;\cos\;\Phi +{\cos\;\delta }_{s}\;\cos\;\alpha \;{\cos\;\tau }_{s}\;\cos\;\Phi +{\sin\;\delta }_{s}\;\cos\;\alpha \;\sin\;\Phi$$

whereas $\delta_{s}$ is declination the angle between equatorial plane and direction of Sun, Ω is azimuth of normal to plane measured clockwise from earth, a is angle between plane and Earth’s surface, Φ is geographical latitude and $\tau_{s}$ is hour angle which is donated by as following Eq. (5);

(5) $$\tau_{s}=\left(12-u\right)15{}^{\circ}$$

whereas u is time of day in 24-h notation, the hour angle is changes at a rate of 15°/h

### 3.3 Determination of the position of the sun

In the case of the horizontal plan (a = 0°) solar elevation angle βs can be calculated from following Eq. (6);

(6) $${\cos\;\theta }_{s}=-{\cos\;\delta }_{s}{\cos\;\tau }_{s}\;\cos\;\Phi +{\sin\;\delta }_{s}\;\sin\;\Phi =\sin\;\beta s$$

The solar azimuth angle (a_s_) defined the direction of the Sun as following Eq. (7);

(7) $$\cos\;\alpha_{s}=\frac{\sin\;\delta_{s}-\sin\;\beta_{s}\;\sin\;\Phi }{\cos\;\beta_{s}\;\cos\;\Phi }$$

$$if\;T_{s}>0\;\left(u<12,in\;the\;morning\right)\;\to a_{s}<180{}^{\circ}$$

$$if\;T_{s}<0\;\left(u>12,in\;the\;afternoon\right)\;\to a_{s}>180{}^{\circ}$$

### 3.4 Numerical solving using Finite Element Method (FEM)

(8) $$K\;T+C\;T_{t}=F$$

In Eq. (8) K is thermal conductivity matrix, T is nodal temperature vector, C is heat capacity matrix, T_t_ is time derivative of temperature and F is vector of external actions.

## 4 Computation and discussion

**Fig. 9:**
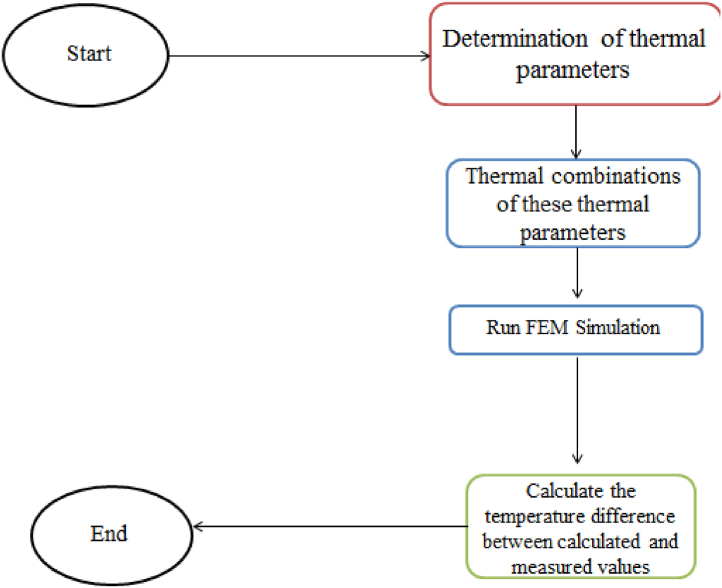
Computational flow chart.

The proposed complex wireless sensors model (CWSM) for real time monitoring of dam temperature is suitable model for real time thermal analysis. CWSM will be used for measuring the effect of temperature, humidity and water pressure on the dam. CWSM use different type of super sensor for real time monitoring of temperature. For this purpose Temperature Sensor, Humidity Sensor, Sun Sensor, Pressure Sensor and Solar Irradiance Sensor are used. Following is CWSM algorithm and flow chart for computation and thermal analysis as in Fig. 9.

**Step 1**: Start process
**Step 2**: Determine the thermal parameter
**Step 3**: Design the combination of thermal parameter
**Step 4**: FEM Simulation
**Step 5**: Input temperature information: T(t) [As temperature is observed and measured with respect to time]
**Step 6**: Calculate the temperature difference between calculated and measured values
**Step 7**: Get output and draw graph for analysis
**Step 9**: End process

**Fig. 10:**
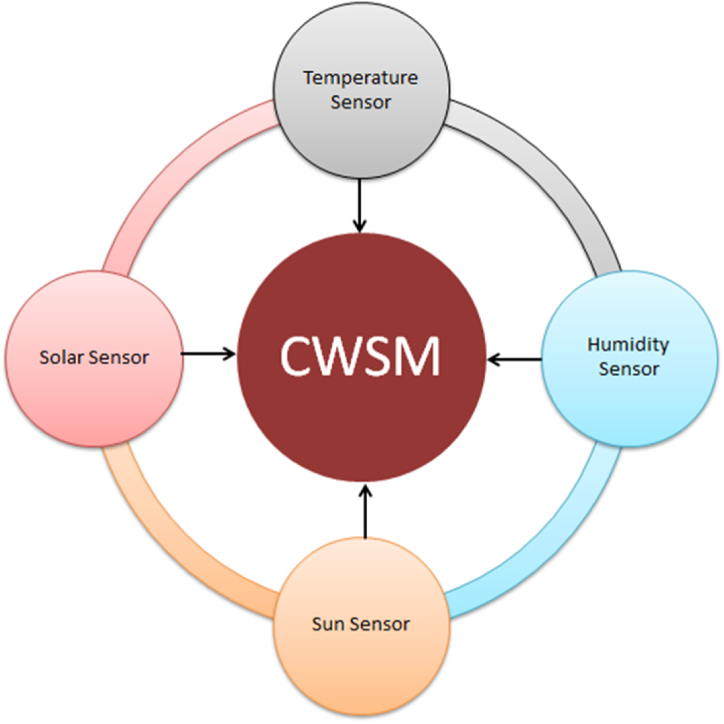
Data collection.

CWSM is based on Token Ring with respect to networking topology. The data is collected from all attached sensors for the FEM thermal analysis as showing in Fig. 10.

## 5 Concluding remarks

Real time monitoring of dam temperature is necessary to conduct thermal analysis. Thermal analysis is performed to minimize cracks occurrence in dams. The cracks occur in dam’s body due to thermal changes. During construction and operation phases, temperature effects on dam are studied. For thermal analysis during construction phase, with respect to transfer of heat, heat of hydration, ambient temperature variation and variation of solar radiations and taking into consideration along with other factors along with cooling pipes. Whereas during operation phase, analytical equations are used for temperature and thermal stresses. So the real time temperate measurement is necessary from engineering point of view for the accurate and timely thermal analysis of dam to avoid cracks and complications.

Keep in view the importance of real time temperature measurement; this paper presents a complex wireless sensors model (CWSM) for real time monitoring of dam temperature. This proposed model is based on the wireless sensors which will be used for measuring the effect of temperature, humidity and water pressure on the dam. The model is also consisting of the sun sensor for observing the elevation of sun. In complex wireless sensors model installation, all the sensors are installed at specific distance. For example the internal side of dam contains two types of sensor i.e. temperature and pressure. Through these sensors, we want to find the temperature and pressure effects of reservoir on the dam. Similarly, temperature, humidity and solar irradiance sensors are implanted at the top of the dam. The temperature sensors will be used to observe the temperature effects of sun on the dam. Moreover, humidity sensors will be used for humidity effects and sun sensors to measure the sun elevation. After the real time measurement of values, 3-D Finite Element method is used for thermal analysis. As this is a prototype model for real time monitoring of dam temperature. So, the statistical analysis of the proposed model will be done in future research.

## Declarations

### Author contribution statement

Jamil Afzal, Zhou Yihong, Usama Afzal, Muhammad Aslam: Conceived and designed the experiment; Performed the experiment; Analyzed and interpreted the data; Contributed reagents, materials, analysis tools or data; Wrote the paper.

### Funding statement

This work was supported by the China Three Gorges University Research and Innovation Fund. This research did not receive any specific grant from funding agencies in the public, commercial, or not-for-profit sectors.

### Data availability statement

Data will be made available on request.

### Declaration of interest’s statement

The authors declare no conflict of interest.
